# Are These Cats Playing? A Closer Look at Social Play in Cats and Proposal for a Psychobiological Approach and Standard Terminology

**DOI:** 10.3389/fvets.2021.712310

**Published:** 2021-07-23

**Authors:** Noema Gajdoš Kmecová, Barbara Pet'ková, Jana Kottferová, Lenka Skurková, Daniel S. Mills

**Affiliations:** ^1^Workplace of Applied Ethology and Professional Ethics, Department of Public Veterinary Medicine and Animal Welfare, University of Veterinary Medicine and Pharmacy in Košice, Košice, Slovakia; ^2^Applied Research Centre, University of Veterinary Medicine and Pharmacy in Košice, Košice, Slovakia; ^3^Animal Behaviour, Cognition and Welfare Group, School of Life Sciences, University of Lincoln, Lincoln, United Kingdom

**Keywords:** cat, intercat, interaction, play, psychobiological approach, social, taxonomy

## Abstract

Play in domestic cats has been largely studied using a contextual approach, i.e., with a focus on what the cat is playing with, such as an object, itself or another cat. Such classification may be superficially attractive scientifically but it limits the ability to investigate function. We propose consideration of a psychobiological approach, which increases attention on hypotheses about the motivational and emotional state of the actors, may be more valuable. This may be particularly important in the case of intercat exchanges that might involve play, for example when one cat may chase another which does not want to be chased, the general interaction should not be considered playful. Key to improving the scientific study of such interactions is the need to adopt a common terminology, thus we synthesise a common ethogram from the published literature. Secondly at the heart of a psychobiological approach is a consideration of both the affective state and motivational goal of each actor in an interaction, since they may not be congruent, and recognition of the hypothetical nature of any such functional classification. However, this bottom up approach provides valuable insights that can be tested. We argue that when one cat treats another as an object or prey, such activity relates to the former cat seeking to learn about its own skills in relation to manipulating its physical environment (prey are not considered part of the complex social relationships and thus social environment of an individual). However, when interaction between cats is reciprocal it may function to facilitate social learning and may be best described as mutual social play. It needs to be recognised that interactions are dynamic and thus our classification of a situation needs to be flexible. So mutual social play may turn into a form of non-reciprocal interaction. We conclude by outlining priorities for future research to help us improve our ability to answer the question “Are these cats playing?” in a wider range of contexts.

## Introduction

Play varies greatly between species but also individuals, and has been examined from a variety of scientific perspectives, so it is not surprising that it has been variously defined. The first comprehensive text on animal play (1) divided it into nine categories: Experimentation, Movement play, Hunting play, Fighting play, Love play, Constructive arts, Nursing play, Imitative play, and Curiosity. Within the “Fighting play” category it was stated that “tussling among animals” could function to practise predatory instincts. Broadly speaking it is widely acknowledged that play can function for motor training, cognitive training and socialisation (2). Focusing on possible proximate functions of play, i.e., the consequences of the behaviour patterns which are of immediate (proximate) benefit to an individual (3), play was considered a “*behaviour that functions to develop, practice, or maintain physical or cognitive abilities and social relationships, including both tactics and strategies by varying, repeating, and/or recombining already functional subsequences of behaviour outside their primary context. It is a matter of taste whether behaviours that do not simultaneously satisfy the structural, causal, contextual, functional, and developmental criteria of this definition are to be called play*” (4).

By contrast, an ethological perspective might emphasise structural aspects suggesting the ultimate function of play. For example, Martin and Caro (3) modified the definition of play by Bekoff and Byers (2) to “*all motor activity performed postnatally that appears to an observer to have no obvious immediate benefits for the player, in which motor patterns resembling those used in serious functional contexts may be used in modified form. The motor acts constituting play may have some or all of the following structural characteristics: exaggeration of movements, repetition of motor acts, and fragmentation, or disordering of sequences of motor acts*.”

Burghardt (5) in his extensive review of the history of attempts to define play instead of trying to create a new definition, proposed a list of five criteria which need to be satisfied in “*at least one respect, in order to identify a behaviour as play in whatever context or species being studied*.” Using this approach, he suggests (5) that play can be recognised as behaviour which is

1. not fully functional in the form or context in which it is expressed;
2. spontaneous, voluntary, intentional, pleasurable, rewarding, reinforcing, or autotelic (for its own sake);
3. structurally or temporally different from strictly functional behaviour expressions;
4. repeated in similar but not rigidly stereotyped form during a portion of the animal's ontogeny;
5. initiated when the animal is in a “relaxed field”—fed, healthy and free from stress or intense competing systems.

This approach has been used to potentially recognise play behaviour in lizards, turtles, bony fishes, stingrays, octopus (6) and even wasps (7). Burghardt's fifth criterion suggests play can be a good welfare indicator, and this has been supported in several welfare related reviews (8, 9).

However, confirming Burghardt's five criteria from field observations can be difficult, especially in a species like the cat. This is evident from the attempted operationalisation of cat play behaviours in a recent review (10) on the development and functions of cat play. Their overview ethogram demonstrates the diversity of descriptions used in cat play studies including contextual, functional but also circular definitions (where “play” is defined as “play”). Therefore, in this review we critically evaluate the classifications used to describe play involving cats, with a particular focus on play *between* cats; on this basis we propose a framework to aid the differentiation of psychobiologically meaningful categories of play and the associated evidence for this.

## Classification of Cat Play

### Challenges From Contextual Classifications

Contextual classifications focus on the circumstances in which play occur in order to define different forms. These are perhaps most widely used with division into locomotor, object and social play (3, 6, 11, 12); however, the distinction between these can be deceptively difficult to define. Martin and Bateson (13) defined locomotor play in cats as activity distinct from manipulation with objects and not directed to other individuals, but rather directed toward the external environment. However, terms such as “self play,” including bouts of a cat chasing its own tail (14) and behaviour that does not appear to be social or directed to an object (15) have also been used to describe play where there is no obvious environmental target. Thus, most definitions seem to agree that locomotor play is usually a solitary activity (6), but not what the target of the action is, nor whether the individual may employ others as “objects” within play. Indeed, the proposed standardised ethogram for *Felidae* delineates this activity as solitary in situations where cat is alone but behaviour patterns such as chasing, pawing, pouncing can be directed to an object or tail of a cat (16).

Object play is typically recognised when an animal manipulates an object and this activity seems to provide no immediate benefit for an individual (6). It has also been referred to using the term “object contact” [pats and paws directed to an object and bites of these objects (17)]. Moreover in cats, play with live prey has been referred as predatory play and differentiated from predation involving non-hurtful manipulation of prey (18). Despite similarity of behaviour patterns in both object and predatory play, there is no consensus on how they should be categorised; for example Mendoza and Ramirez (19) differentiated predatory play from two other subcategories, which they referred to as social and non-social play (which included object and self-directed play).

Whether or not a classification is actually *contextual*, may also be confusing. For example, the term “social play” can merely describe playful activity directed toward a conspecific (19), but in other contexts it may be applied to describe behaviour that has a particular emotional-motivational basis (20). In this latter context a cat who “plays” with another and treats it like a prey object is not engaged in social play but rather a “SEEKING” [*sensu* (20)] type of activity.

Contextual classifications may be superficially attractive but they appear to be often arbitrary and do not inform about motivation, having little biological relevance. Thus, they are not very useful clinically when considering how to manage these responses if they are seen as problematic. Managing a cat who is perhaps more predatory in its playful actions toward another cat requires quite different intervention to one who is engaged in rough and tumble play. In the latter the both might be in a positive affective state, but in the former the one being chased (if the behaviour is not reciprocated) could be in a very negative affective state.

### Functional Classification

Operationalised definition of cat play (10) highlights that “social play” is probably one of the most frequently used but also most variedly defined terms. It may be simply defined as play directed to conspecifics (6) but might also include play with a human (21, 22); it might also be associated with activity directed toward a toy by more than one individual at a time: “*activity of two cats playing with same toy simultaneously or within 3s*” (23). Beyond the aforementioned definitions, it has been argued that “common sense” be used to recognise social play in cats. In one of the earliest studies of play in kittens, the authors admit that the observer usually has an intuitive sense for recognising playful behaviour and they used working definition criteria only in occasions where the playful character of interactions was not obvious (17). However, this obviously poses challenges when we consider the scientific quality of the work (such as its potential replicability). Indeed in this latter study, (17) authors admit that despite applying these criteria and excluding harmful interactions, in older animals they may have scored as playful some interactions that might have been “serious.” The risk of recording “serious” interaction as play becomes even greater when very broad definitions are used, e.g., all social encounters between cats are considered to involve social play (24, 25).

Likewise, referring to a playful activity in terms of specific behaviour patterns (e.g., chasing or biting) supplemented by adjectives such as “friendly” and thus relying on subjective assumption that they are not agonistic (26, 27) may be similarly problematic. This highlights the difficulty of distinguishing play from agonistic interaction, especially in a species such as the cat. Relying on descriptions based on circumstances, combined with common sense or subjective beliefs is not sufficiently scientifically robust for recognising play. An alternative approach is to begin by acknowledging that the labelling of something as play involves making an inference about it, which inherently implies there is some uncertainty about the accuracy of this. Thus, the description of play is a postulate that needs to be supported by several lines of evidence, but can still be subject to potential scientific falsification as new evidence comes to light.

Within the field setting, it has been argued (28) that it is useful to differentiate three elements to a behaviour, its contextual, motivational, and emotional basis. Context (the circumstances surrounding expression of the behaviour), can be defined objectively, however both its motivational (biological goal) and emotional (personal significance) basis cannot be measured directly (28) but can be inferred with varying degree of confidence by triangulating the evidence available from careful and systematic observation of the antecedents to the behaviour and its consequences (motivation) and the stimulus contingencies, signs of arousal, behavioural tendencies, and communicative signals (emotion). With this approach, it is recognised that the description of an action as playful remains a hypothesis that can be tested (and potentially falsified in line with scientific methodological requirements), for which the evidence can be gathered objectively. This approach has the potential to link the behaviour with a meaningful psychobiological basis. For example, when the term social play is used purely in relation to context, i.e., it is play occurring between two individuals (6, 11), this tells us nothing about underlying mechanism in terms of neurobiology or psychological state. Panksepp (20) argues that from an affective neurobiological perspective what he describes as social play (PLAY) is a pleasurable reciprocal interaction (rough and tumble play) that affords both individuals the opportunity to obtain important social skills which can be used later on in life. We propose below, that the term “*mutual social play*” is a preferable term as it emphasises not only the context (a mutual interaction) but also the motivation (social play) and potentially its emotional quality (the social pleasure associated with PLAY).

From this perspective, what is described as object play, locomotor, and self-play are also pleasurable activities but lack the social dimension; they have the potential function for the individual to not only learn about the physics of their environment, including both animate and inanimate objects, and potentially awareness of their own body; but also to facilitate the development of future behavioural skills.

Behavioural similarities between object and predatory play suggest common motivational elements; clearly there is also a close relationship between these motivational systems and those involving feeding including its natural precursor: predatory behaviour. For example, hunger motivates cats to interact with larger toys which are otherwise neglected (29), but it also leads to the performance of apparently playful behaviours with large prey such as rats (18). It might be that these associations are the product of related, but separate, functional motivational systems regulated by a common affective system [SEEKING *sensu* (20)]. However, an alternative psychobiological perspective might suggest that the functional relationship between these activities is even closer, than outlined. It has long been argued, that what is often called “predatory play” may be a misperception of inhibited predatory behaviour and not related to a separate play motivational system (30). It is suggested (30) that the perception of “play” comes from a failure to consummate the predatory action with a kill, and the seemingly exaggerated actions directed at the prey; however this might instead reflect an emotional tension between a desire to attack and an anxiety to avoid potential harm from the prey (30), within a single functional predatory system. The seemingly exaggerated playful behaviours, might be functionally important in avoiding harmful contact from the prey (30).

The psychobiological perspective may thus help address Burghardt's argument (6) that play is not fully functional and the classification of play should not focus solely on function. We suggest that a primary focus on the underlying qualitative emotional state of the individual engaged in apparently playful behaviour, alongside consideration of the functional behavioural systems that might be involved and how they develop, provides valuable insight into the problem of the classification of play in cats (and other species). This is particularly useful when considering the factors influencing the different forms of play described in the literature and how they might be most effectively managed (31).

**Figure 1:**
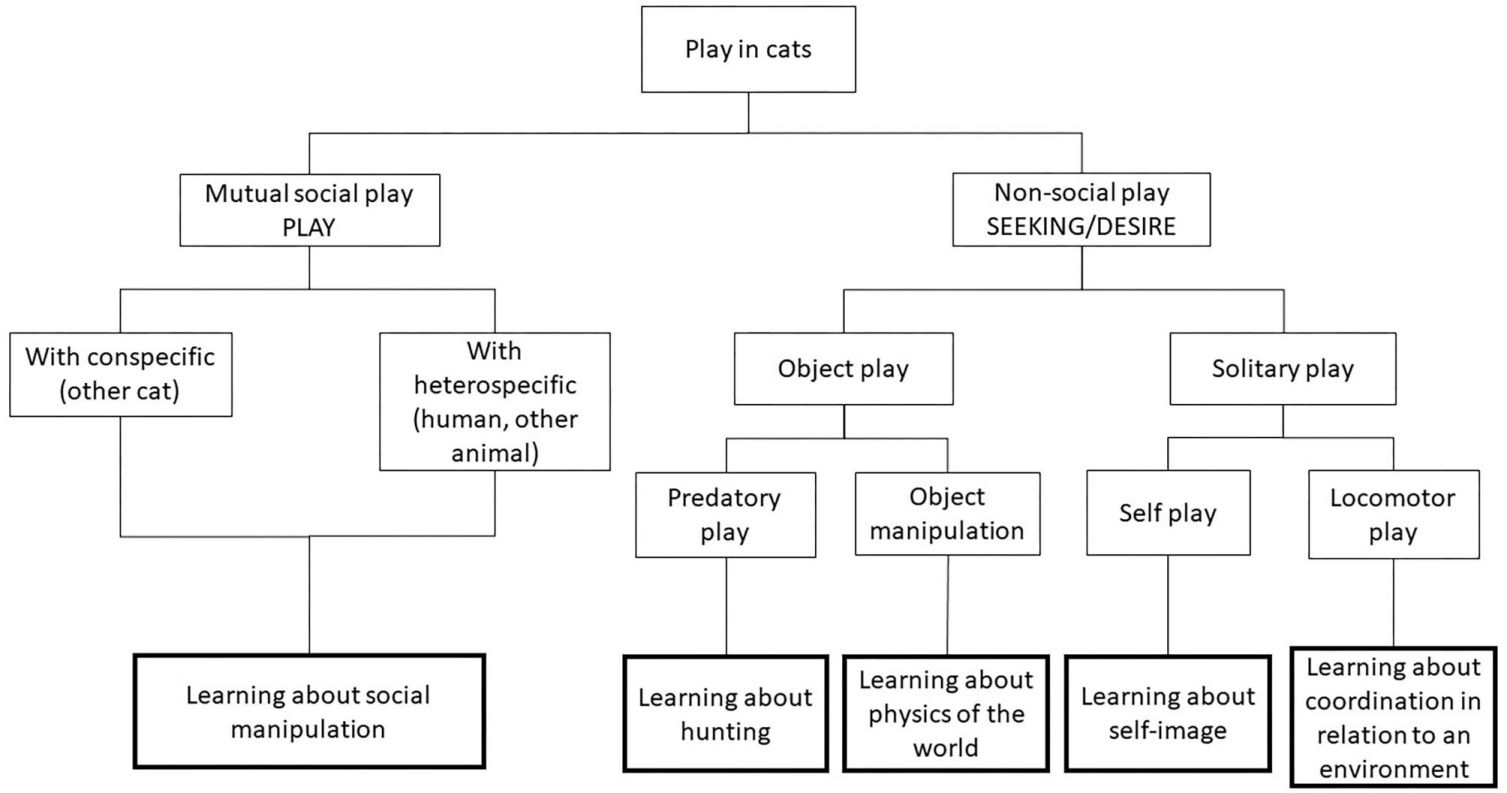
Proposed psychobiological classification of play in cats. At the first level the affective (psychological) basis to the type of behaviour involving play is distinguished using the terminology of Panksepp (20). The lowest level represents the specific functional (biological motivational) goals of specific types of action, several of which may be related to a given affective system.

This psychobiological framework (Figure 1) places affect at the top of the mechanistic considerations and is able to embrace the diversity of play seen both with and without another individual in a rationally consistent way. For example, when one cat is playfully hunting or manipulating the tail of another, we would argue this cat is not engaged in mutual social play, but rather some form of object play, which is related to the affective system described by Panksepp (20) as SEEKING. Likewise, when interaction is not reciprocal and one cat is treated by another cat as if it was a prey or object, the necessary criteria for social play (from a psychobiological perspective) are not met as it is not a *reciprocal* pleasurable or mutual activity, and so it should be classed as a separate type of activity.

**Figure 2:**
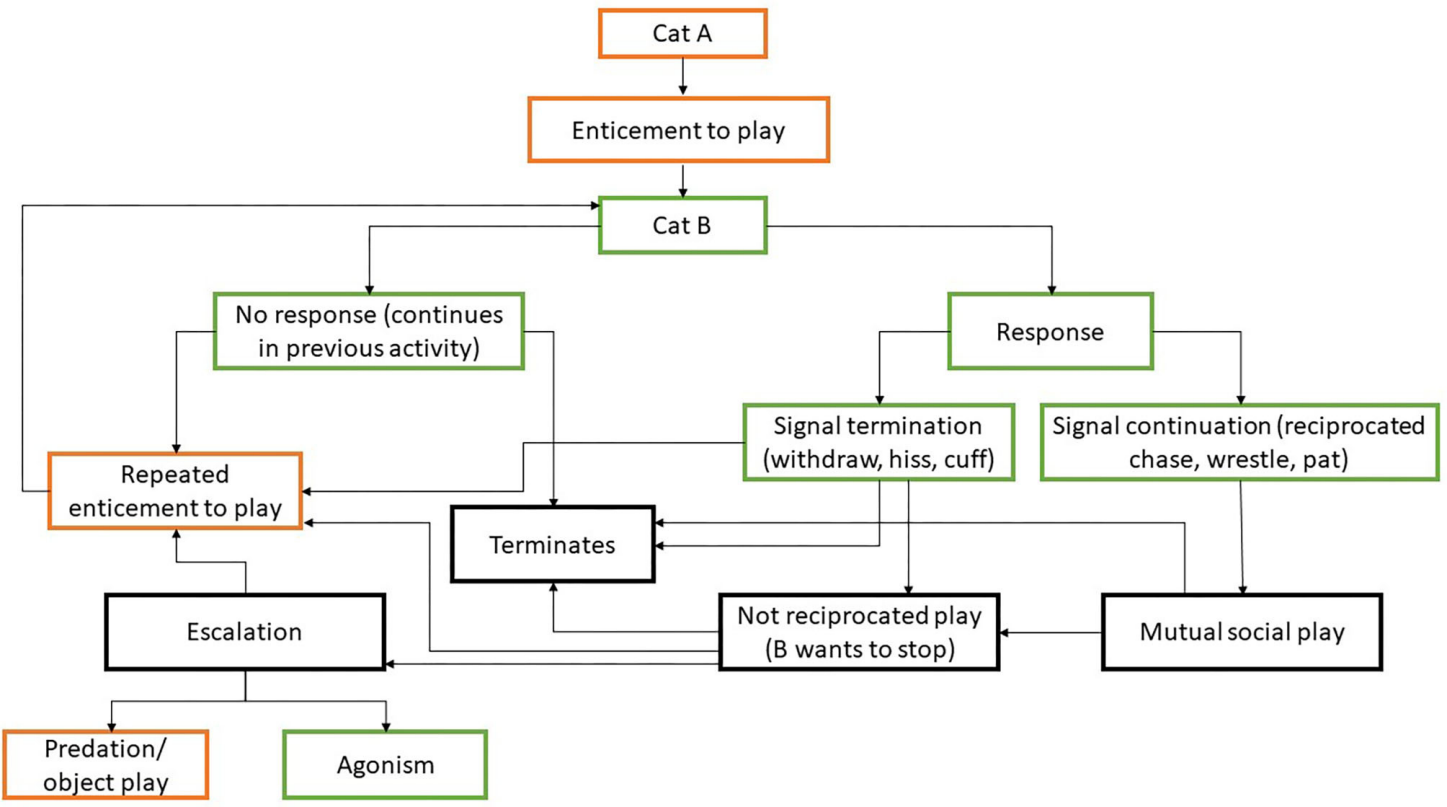
The elements intricately linked with intercat play (including elements of mutual intercat play). Any element could be considered part of play, due to context but motivationally may involve other systems [inspired by diagram of the sequence of interactions in black bear social play (6)].

This approach also helps to highlight a number of important practical considerations. For example, as cats differ greatly in their social requirements, e.g., contact with conspecifics may be beneficial for one cat but might be stressful for another (32): social interaction, including apparently playful activity, does not necessarily support good welfare (33); instead we need to consider the specific emotional predispositions of the individuals involved and thus what is important to them as individuals. This focus on underlying affect, also highlights the potential for meaningful change within a given interaction. Thus, an interaction between cats may start off as a form of mutual social play but develop into something quite different. If the play stops being reciprocal and/or one cat wants to terminate the interaction, e.g., by trying to escape after a bout of mutual chasing (34), the response of the other cat is critical to how what follows should be viewed psychobiologically. It may accept this and stop interaction (will not approach the cat which left after the chase), or it may entice the individual to play again by pouncing on the cat that left the interaction (for the definitions of the behaviours see Table 1) (34), which might result in withdrawal (the cat walks away again), aggressive behaviour (bite) (41) or a reciprocal response in this cat (pounce) leading to the resumption of mutual social play. We illustrate these potential sequences within the context of intercat exchanges and their interpretation below in Figure 2.

**Table 1:** Overview ethogram of intercat play behaviours with suggested common terms of the variables.

**Ethogram element as per Stanton et al. (16) unless otherwise indicated (highlighted in bold)**	**Description**	**Equivalent term used by other authors**	**Equivalent definition used by other authors**	**References**
Approach 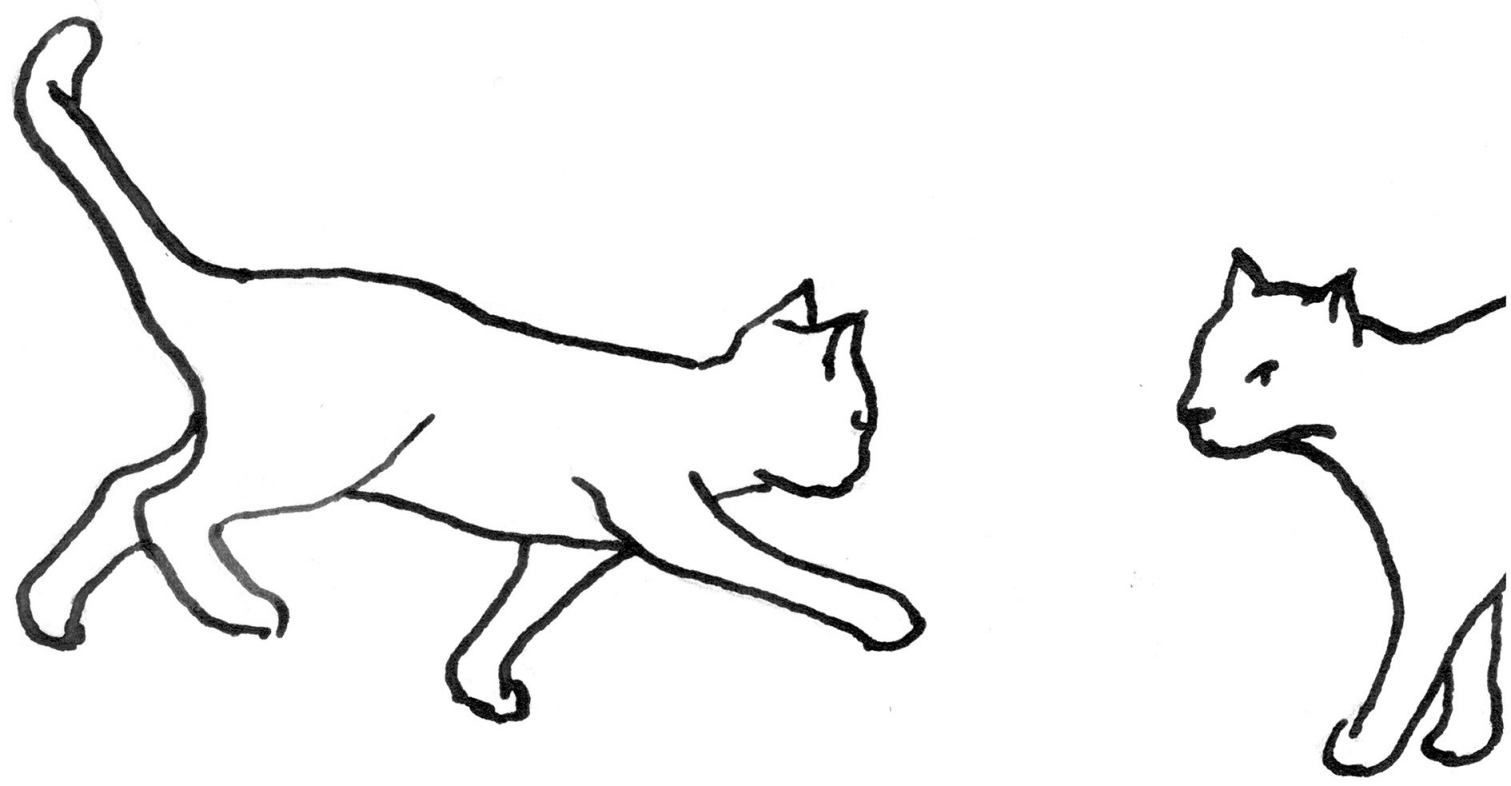	Cat moves toward cat while looking at it.	Approach	Locomotion of any sort toward prey/sibling.	(24, 35)
		Approach	Movement of any sort (excluding canter) toward another cat.	(25)
		Approach	Each occurrence of movement by an individual from at least two kitten body lengths away from another individual to less than two kitten body lengths away from that individual.	(14)
Arch back 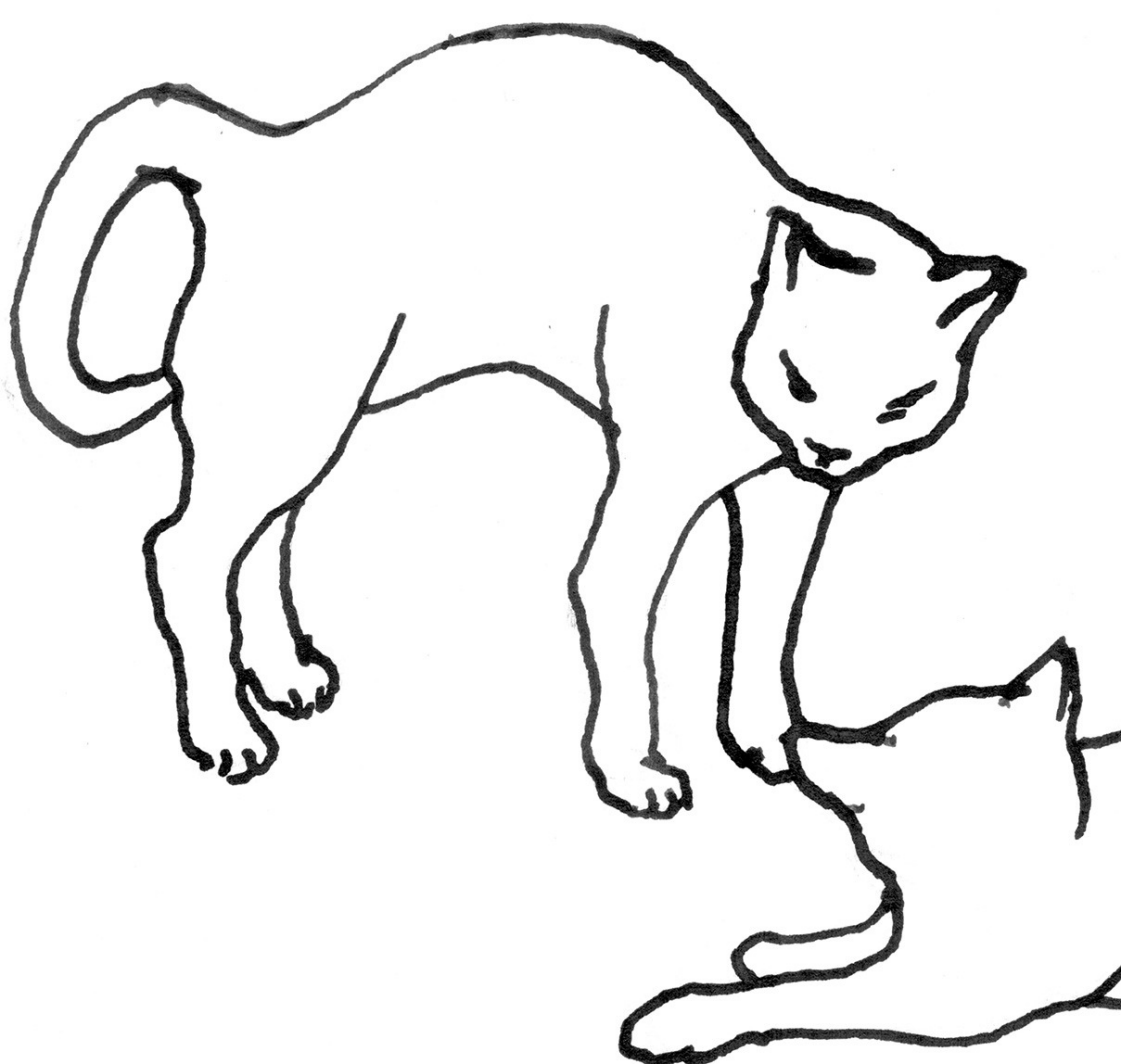	Cat curves back upwards and stands rigidly.	Horizontal leap	The kitten assumes a lateral position, with respect to another kitten, arches back slightly and curves its tail upwards and toward its body then leaps off the ground.	(34)
		Side-step	The kitten arches its back, curls its tail upwards and walks sideways toward or around another kitten or object.	(34)
		Arch	Each occurrence of a marked upward curving of the spine while standing still, leaping upwards, or moving sideways. The orientation is usually side-on in relation to another cat or object.	(17, 36)
		Neck Flex	Each occurrence of a marked downward flexion of the neck. The head is also turned to face another cat if the body is side-on. It can occur simultaneously with the Arch and can be given while standing still or moving sideways.	(17)
		Arch	A marked upward bending of the spine while standing still, leaping upwards or moving sideways.	(24, 35)
		Arch	A marked upward bending of the spine while standing still, leaping upwards or moving sideways, with or without piloerection.	(25)
**Belly up** 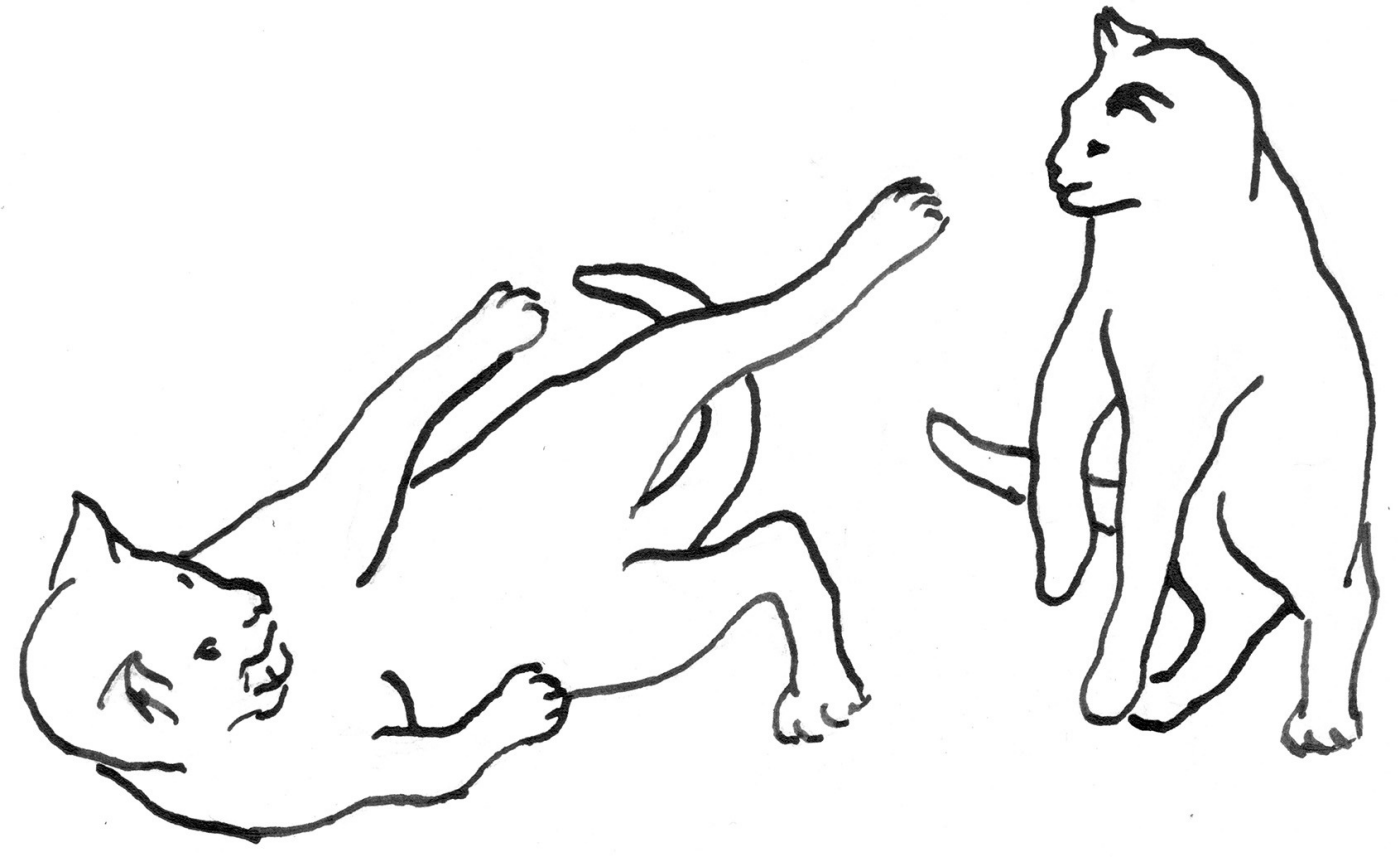	The cat lies on its back with front or all limbs held up but not touching another cat. Back legs may be alternating between flexion and extension and front legs reaching toward another cat which may be standing over the subject cat. The tail is typically straight back and may be moved back and forth. Mouth is held open and teeth are exposed.	Belly-up	The kitten lies on its back, its belly up, with all four limbs held in a semivertical position. The tail is straight back and may be moved back and forth. Typical paw movements associated with the belly-up posture are to move the back legs in a treading motion and to make reaching or pawing movements with the front legs. The mouth is held open and the teeth are exposed. In a social encounter, one kitten assumes the belly-up position and another kitten stands over it. Thus, the treading and pawing movements bring the kitten into contact with parts of the body of the standing kitten. Usually, these areas are the head, neck and ventral area.	(34)
		Mouth open	Gaping at another cat while in a rolled position.	(25)
		Paws up	Front paws, and sometimes back paws as well, held up to but not touching another cat, while subject is in a rolled position.	(25)
Bite 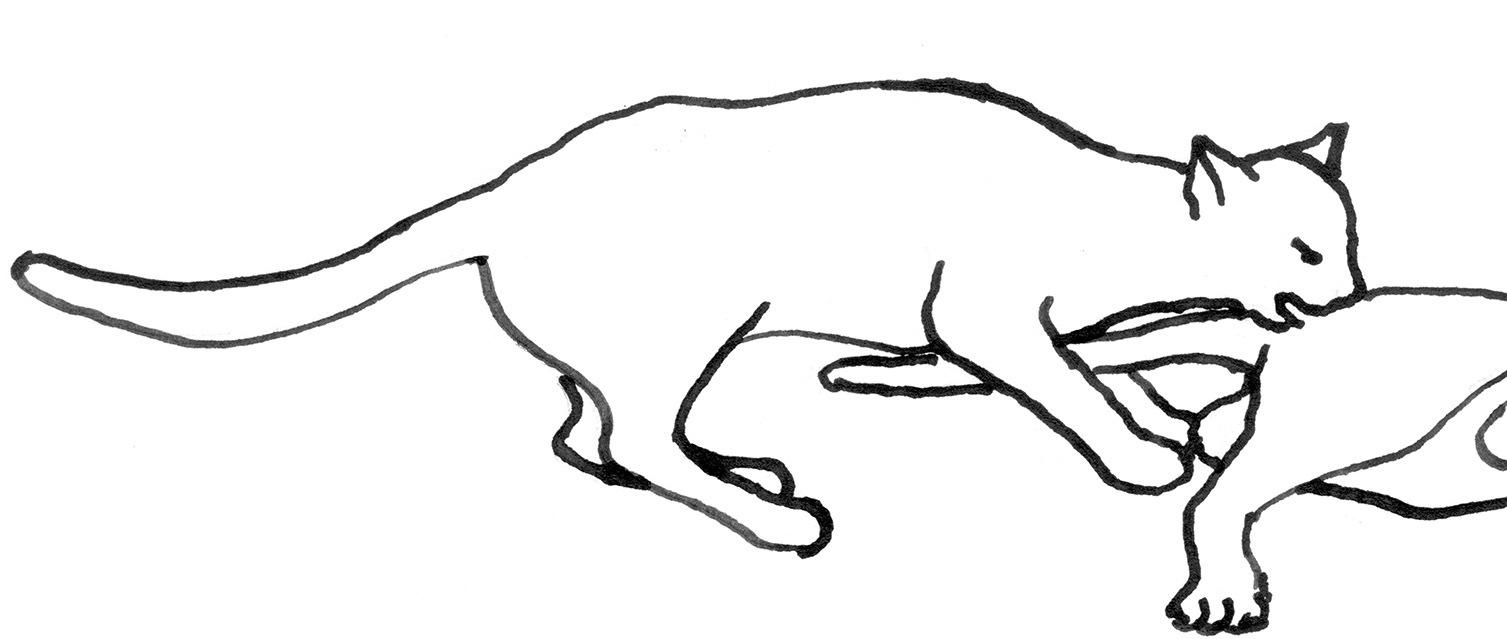	Cat snaps teeth at and is successful in making contact with another cat.	Bite	Bringing jaws into contact with the prey/sibling and closing them.	(24, 35)
		Bite	Bringing jaws into contact with a cat and closing them.	(25)
**Canter** 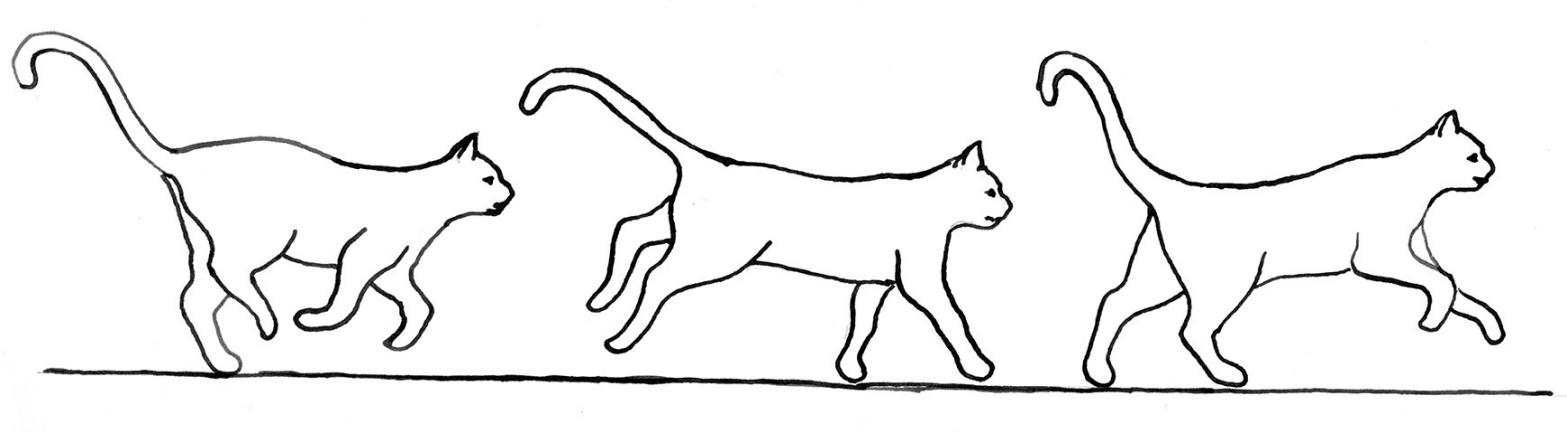	Asymmetrical running gait during which all paws repeatedly and simultaneously leave the ground and limb movements patterns are different on the right and left side; head and tail may be held high.	Canter	Jerky running gait during which all paws repeatedly and simultaneously leave the ground; head and tail often held high.	(25)
Chase 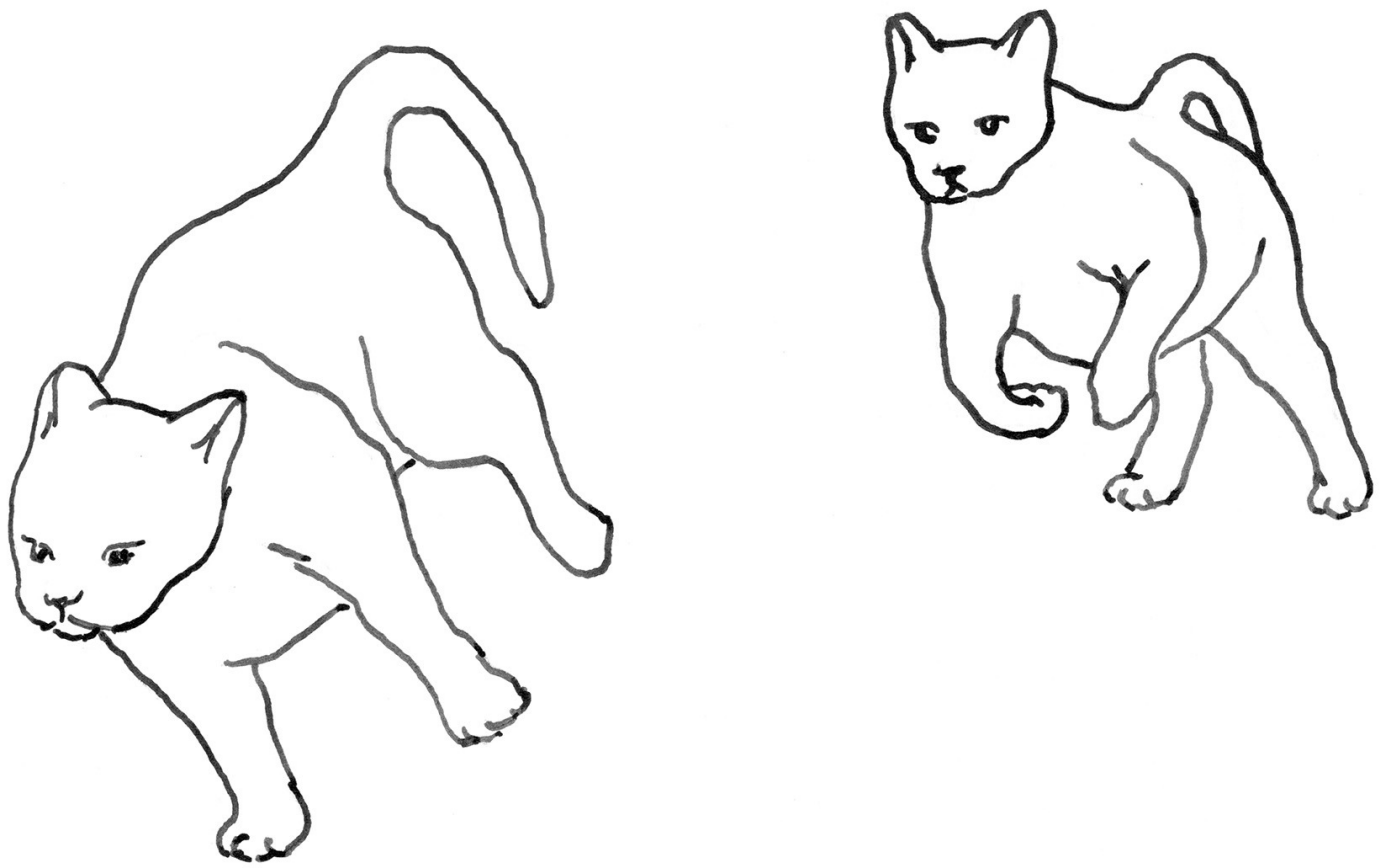	Cat runs rapidly in pursuit of cat.	Chase	A chase involves a kitten running after or from another kitten. It could, perhaps, be differentiated into pursuit and flight.	(34)
		Chase	Running after a moving kitten.	(24)
		Chase	Running after a moving cat.	(25)
		Chase	Each bout of running after another individual/mobile object with the chased individual running away from the chaser for at least a distance of 1m.	(14)
		Flee	Cat runs away from cat.	(16)
		Flee	Running while being followed by a moving cat.	(25)
**Face-off** 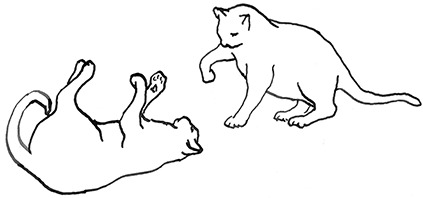	Cat is sitting near to another cat with head and neck oriented toward it and body hunching forward. Cat is moving its tail back and forth and may lift a front paw and move it in direction of another cat. The other cat may be in a similar face-off position or may be in belly-up position (as shown here).	Face-off	A kitten sits near another kitten and hunches its body forward, moving its tail back and forth, and lifts a front paw and moves it in the direction of the other kitten. The kitten's head and eyes are also oriented toward the other kitten. Two kittens may face-off simultaneously and direct their front paw movements at one another's face.	(34)
		Face off	Sitting next to another cat, often with tail lashing and head twisting; recipient in a rolled position or similar face off stance).	(25)
Paw 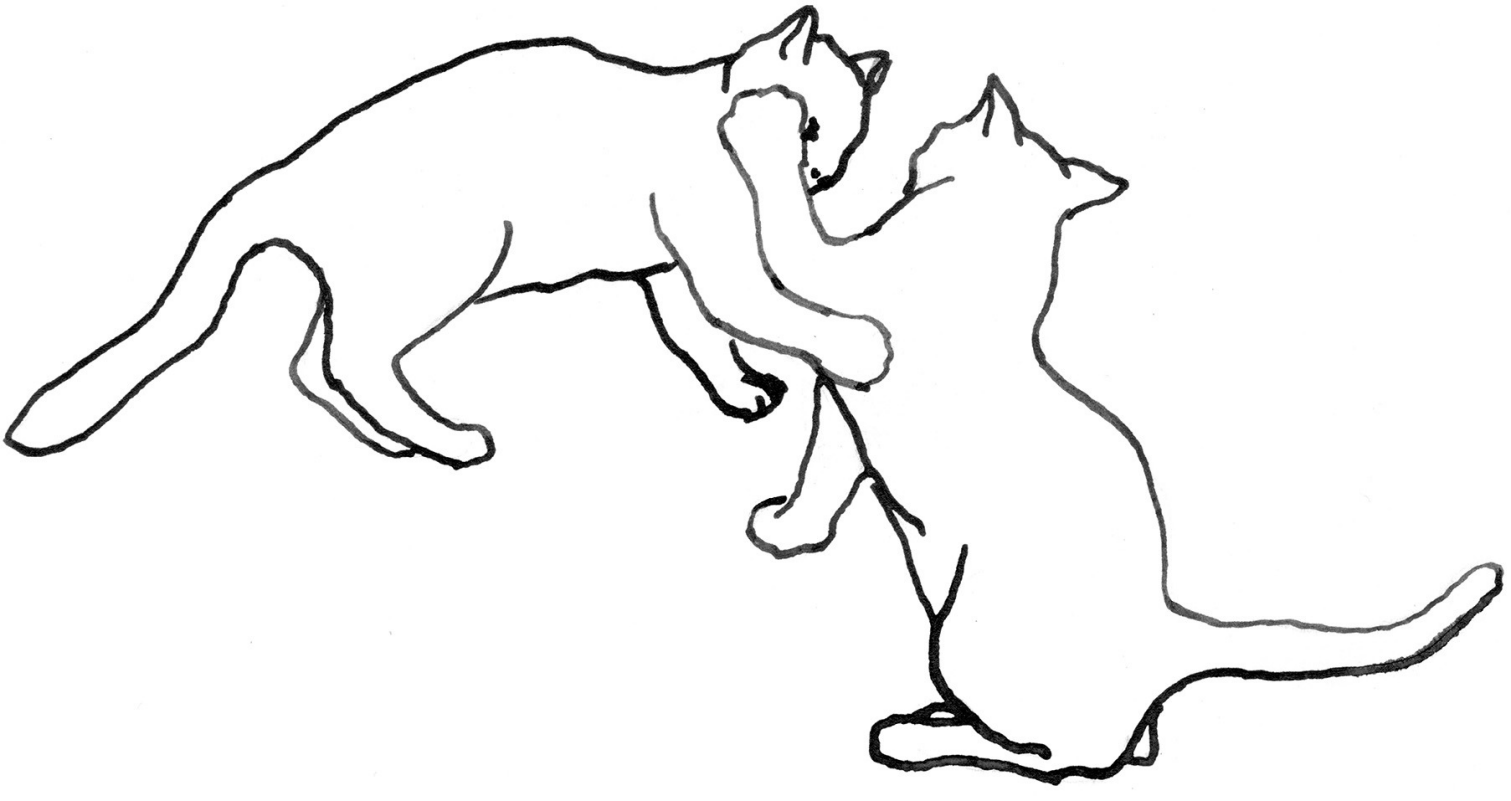	Cat pats cat with its forepaw(s). Claws are usually retracted.	Paw	Bringing the forepaw into contact with the prey/sibling.	(24, 35)
		Paw	Bringing the forepaw into contact with a cat.	(25)
		Cat contact	Includes pats and bites: Each pat with a paw making contact with another cat and each bite of another cat.	(17, 36)
		Cat Contact	Each pat with a forepaw, and each bite, making contact with another cat (mother or sibling).	(37)
		Paw/pat	Each occurrence of a pawing/patting… movement directed at another individual/mobile object which lasts no longer than 1 s and also involves no grasping or holding of the individual/mobile object.	(14)
Pounce 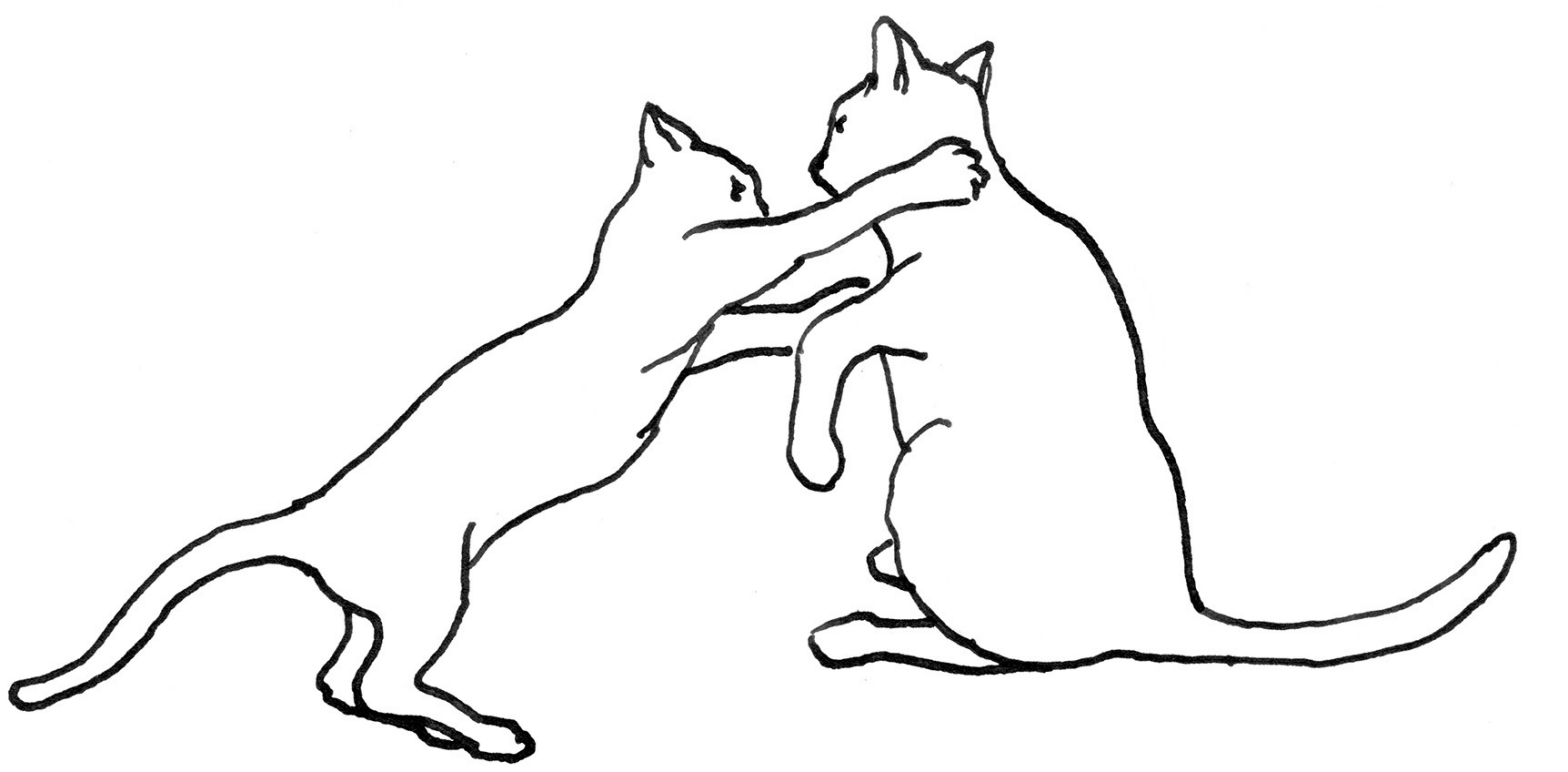	Cat leaps onto cat.	Pounce	The kitten crouches with its head held low or touching the ground and its back legs tucked in and its tail straight back. The tail may be moved back and forth. The kitten moves its hindquarters back and forth and moves forward, the thrust coming from the extension of its back legs.	(34)
		Attack	Jump onto a cat and grasp it with forepaws or forelegs.	(25)
Rear 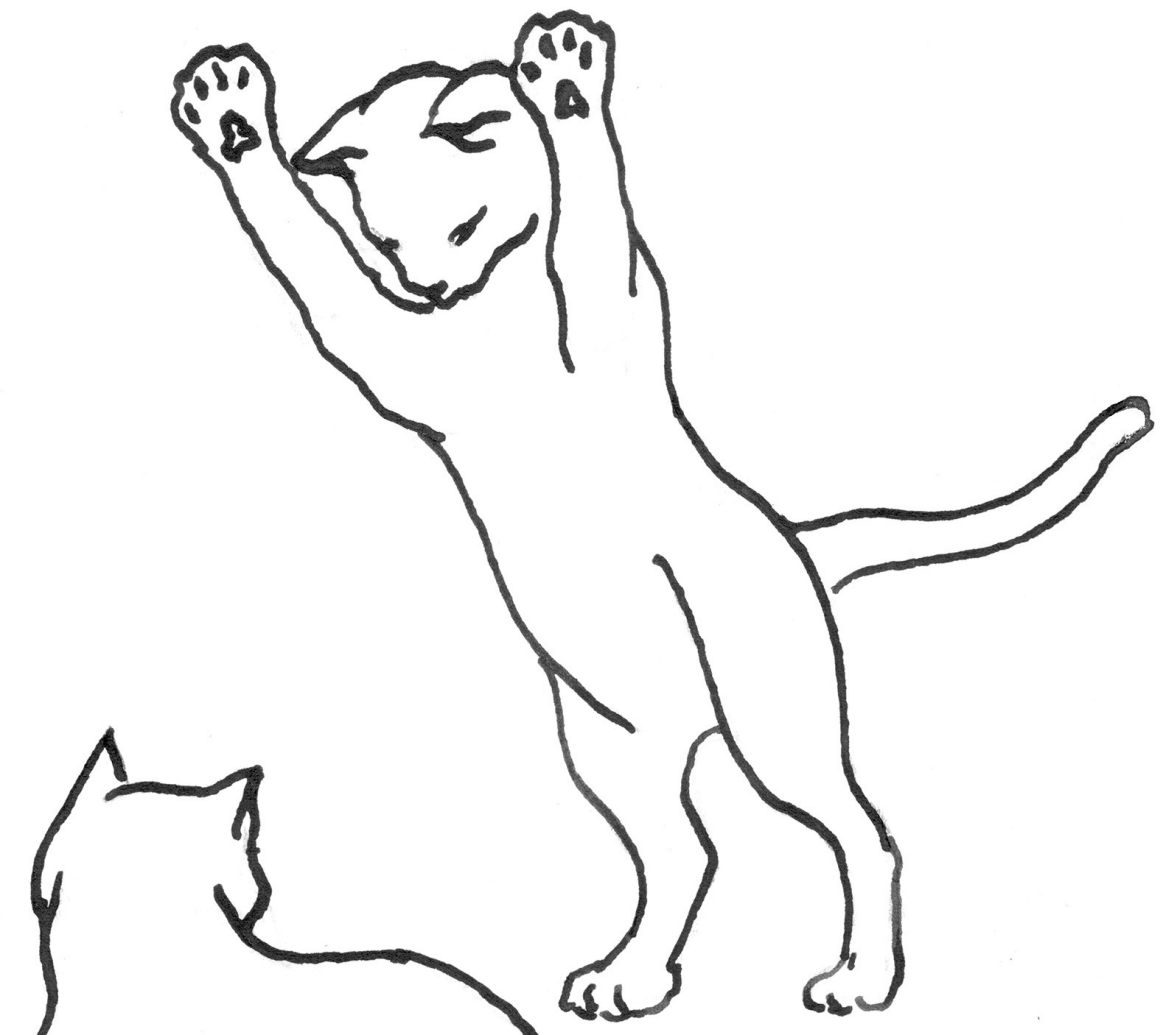	Cat stands up on its hind legs with forelegs toward or against cat.	Vertical stance	A kitten assumes a sitting position and then rocks back on its hindquarters, lifts its front paws off the ground and stretches them out perpendicular to its body. The kitten also extends its back legs so that it is in a stationary bipedal position.	(34)
		Rear	Each occurrence of sitting, standing or vertical leaping on the hindlegs with forelegs raised and splayed. It was performed beside another cat or object.	(17)
		Rear	Standing or vertical leaping on the hindlegs, with forelegs raised and splayed.	(35)
		Rear	Each occurrence of sitting, standing or vertical leaping on the hindlegs with forelegs raised and splayed.	(36)
		Rear	Standing or vertical leaping on hindlegs, with forelegs raised and splayed.	(24, 25)
Stalk 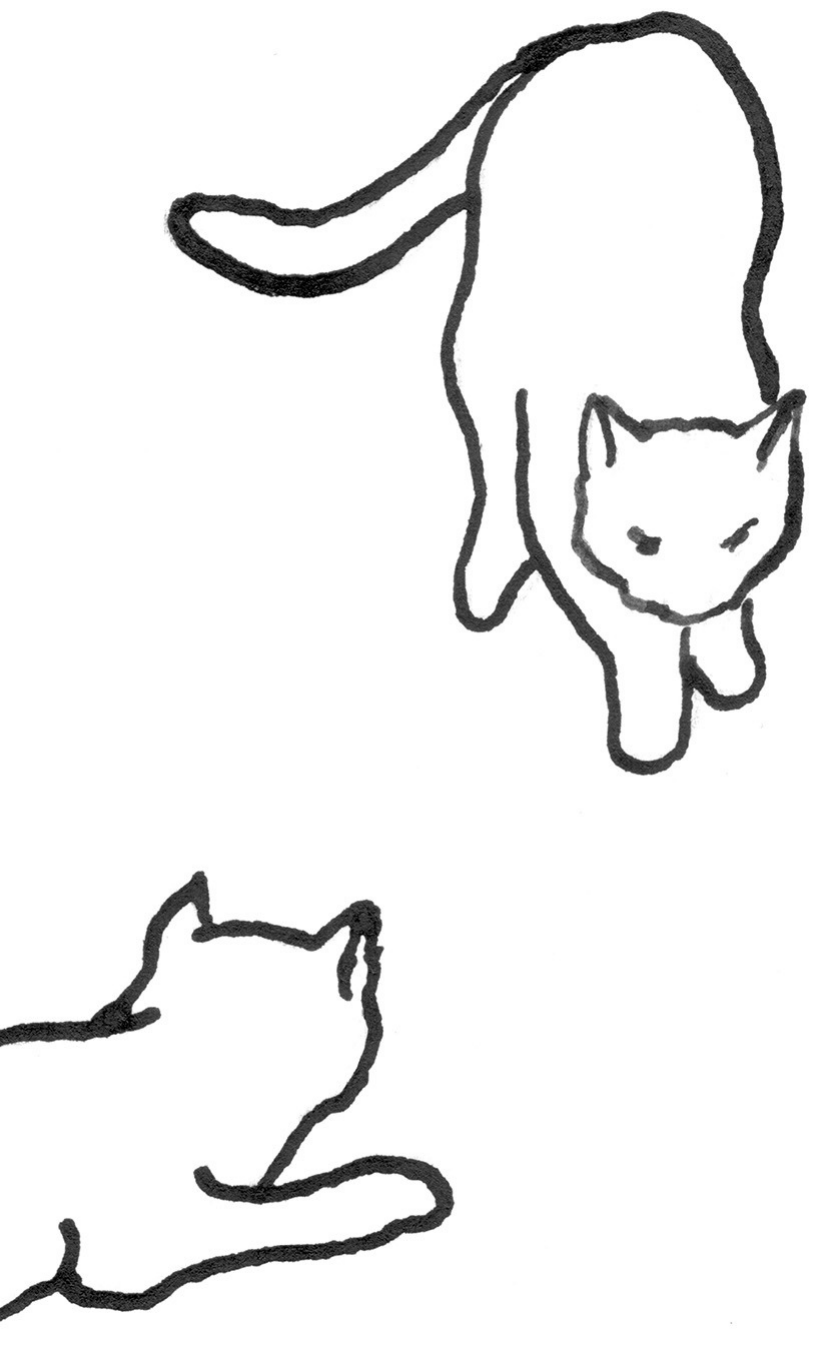	Slow, forward locomotion in a crouched position directed toward cat, with head kept low and eyes focused on cat.	Stalk	Each bout of crouching with hindlegs treading, or creeping (or running briefly) with belly close to the ground and head low toward another cat or object.	(17)
		Stalk	Each bout of low crouching with hindlegs. Treading or creeping (or running briefly) with belly close to the ground and head low toward another cat or object.	(36)
		Crouch	Belly on the ground with all limbs by the side of the body, oriented and attentive to a conspecific; backlegs often treading.	(25)
		Stalk	Each bout of low crouching with hindlegs treading, or creeping (or running briefly) with belly close to the ground and head low toward another individual/mobile object (17).	(14)
**Stand-off** 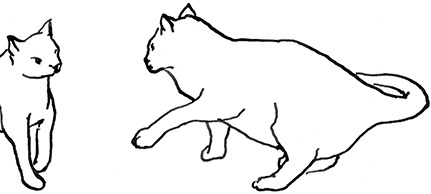	The cat stands near or over another cat with its head oriented toward the head and neck region of the other cat. The subject's mouth can be open and it may raise one of its front paw and paw at other cat.	Stand-up	The kitten stands near or over another kitten with its head oriented toward the head and neck region of the other kitten. The stand-up kitten's mouth is open and it may direct “bites” toward the other kitten. The kitten may also raise one of its front paws and paw at the other kitten.	(34)
		Stand off	Standing next to another cat, often with head twisting; recipient usually in a rolled position.	(25)
Wrestle 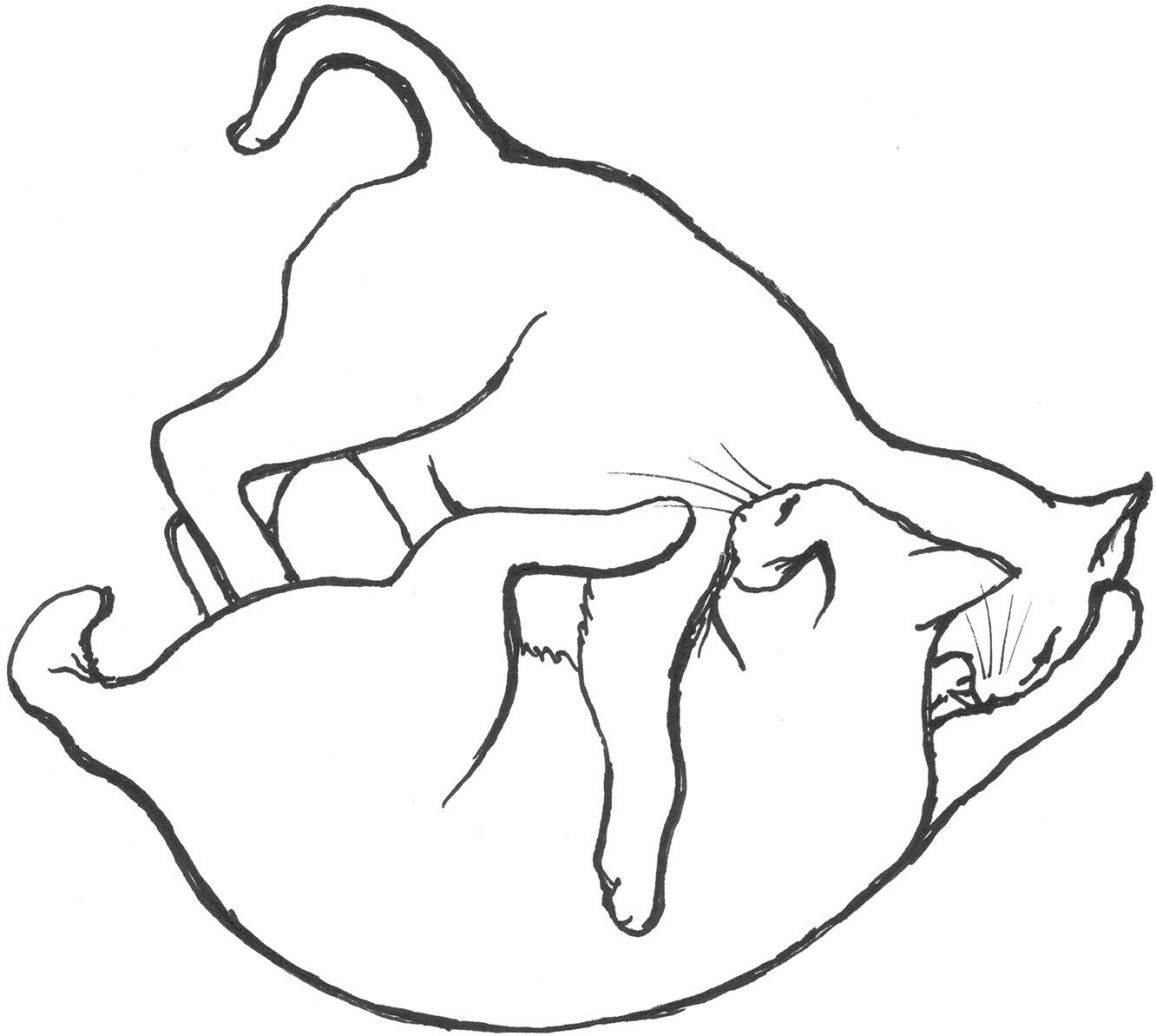	Cat engages in physical contact with cat, whereby the cat struggles with cat. Can include pulling cat toward itself with its forelegs and perform raking movements with the hind legs.	Wrestle	Each bout of lying while clasping with forelegs and kicking with the hind legs another cat or object. This pattern formed part of West's (34) “Belly-up.”	(17)
		Rolled contact	Lying on dorsal or lateral surface and employing any of the above contact patterns. (Contact patterns refer to the combined paw, hold, bite and carry scores).	(35)
		Hold	Bringing the forepaw or forearms simultaneously into contact with the prey/sibling.	(24, 35)
		Hold cat	Each occurrence of grasping another cat between the lower part of the forelimbs.	(36)
		Hold	Bringing forepaws or forearms simultaneously into contact with a cat.	(25)
		Rolled contact	Lying on dorsal or lateral surface and employing any contact pattern (Paw, Hold, Bite); (a similar pattern to Barrett and Bateson's “wrestle”).	(24, 25)
		Four paw contact	All four paws in contact with another cat while subject is in a rolled position.	(25)
		Foot contact	Contacting another cat with one or two back paws or backlegs, e.g., stepping on, kicking once or repeatedly kicking with backlegs in unison.	(25)
		Rake	Each bout of kicking movements at another cat or at an object with one or both hind legs. A component of Barrett and Bateson's (17) “Wrestling.”	(36)
		Roll	Each occurrence of rolling on the side or back [see (38)]. Overlaps with Barrett and Bateson's (17) “Wrestling”	(36)
		Wrestle	Time spent by an individual holding/grabbing another individual/mobile object, sometimes kicking at it with the back legs [incorporates Hold Cat, Hold Object and Rake (36)].	(14)
		Wrestle	One cat struggles with another cat, raking with its hind legs and pulling the “opponent” toward its body with its forelegs. It is mainly a play behaviour, and is distinct from FIGHT (being much less intense and lacking the additional elements of FIGHT).	(39)

*Caption 1 Terms highlighted in bold—No equivalent found in standardised ethogram for the felidae by Stanton et al. (16), therefore term by original authors is used and proposed inclusive definition (2nd column). Pictures are redrawn from (17, 35, 40) or drawn according to the descriptions*.

*Note the ethogram does not include behaviours used to terminate play, since these are generally forms of non-reciprocation or escape by one of the cats. For visualisation purposes, we have used a convention of showing both cats in full, where the behaviour of both actors is important, where it is not, then the cat whose behaviour is not relevant is shown as a partial image*.

Given the psychobiological mechanistic complexity of these scenarios, it is not surprising that besides the scientific confusion that has existed, there is often considerable uncertainty among cat owners concerning the behaviour of their cats' interactions. Consistent terminology and the processes described here for classifying the activity can help determine what is probably happening, but there is undoubtedly a need for greater research and objective data to reduce the uncertainty concerning whether two cats are playing, and the implications of this for their well-being. To this end, we suggest that the term “*intercat play*” be used purely as a contextual description of an interaction which appears playful at some level, with no implication concerning underlying emotion or motivation, nor mutuality. In order to build the necessary evidence base to make the inferences we suggest, it is necessary to have an agreed terminology for the structural behavioural elements of play in the cat. Accordingly, in the next section, we review the ethograms used to describe intercat play and propose a standard terminology for future use.

## The Structure of Intercat Play—A Revised Ethogram

The work of Stanton et al. (16) provides a useful framework for a standard ethogram of intercat play, but we suggest it is incomplete. Therefore, Table 1 is a more comprehensive ethogram based on the available observational studies of intercat play in domestic cats. In order to highlight where there might be confusion, Table 1 also highlights when the same or similar terms are used by other authors but with potentially different definitions.

**Figure 3:**
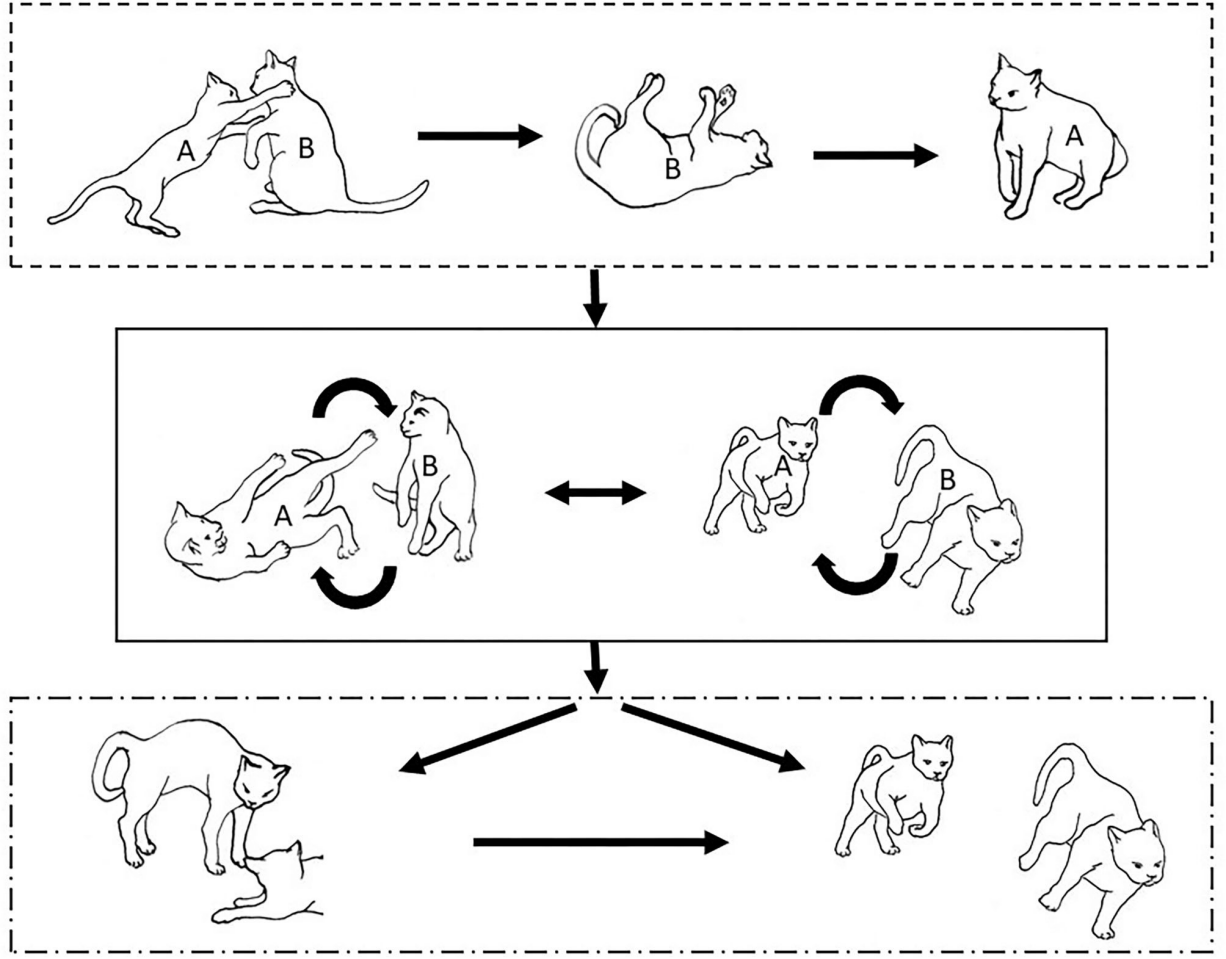
Sequences involved in mutual social play [inspired by (34)]. Top box represents sequences which often form part of initiation of the play, middle box includes those which are seen in continuation of the play and in the bottom box, behaviours which can terminate mutual social play bout without aggressive interaction, are depicted.

On the basis of the study by West (34), who offered a description (rather than true sequence analysis) of behaviours that appear to be potentially part of what we term “mutual social play,” we suggest that this activity is often initiated by one cat pouncing on another who often responds with a belly-up posture. However, a combination of belly up and stand-off posture can also be seen as an initiation pattern for mutual social play. These two patterns—belly-up and stand-off, together with face-off behaviour are regular parts of the continuation of mutual social play, while it is most commonly terminated by chasing and arching. Reciprocity is demonstrated by a combination of certain behaviours such as pouncing with belly-up or rearing and a stand-off posture as a response to the belly-up posture (Figure 3). Alternation of these two behaviours has also been suggested to serve as a signals of playful intent (34). Interestingly, rolling on the back with the abdomen exposed (which is similar to the belly-up posture) has been observed within intercat play by various authors in a range of contexts: in association with “wrestling” behaviour (25, 34, 36, 38); but also within affiliative (26) and agonistic contexts, where it may be interpreted as a potentially deferential, appeasement or submissive gesture (42, 43). Further research is required to establish if it plays a role in the proximate regulation of social play in cats.

The structure of intercat play changes with age, and this may reflect shifts in behavioural maturation and the associated motivational and emotional systems or stimuli influencing the occurrence of the behaviour at any given time (17, 24). Examining the temporal relationships between certain behaviours which have both a specific function and which are also expressed in play may provide important insights into how the importance of certain forms of play may vary with age. For example, side stepping (34) declines as a feature from 12 weeks and this may be because of its resemblance to the defensive arched back posture seen in the agonistic encounters of adult cats. Similarly, the related term “arch” measured in other studies (17, 24) decreases in frequency from the 7th to 12th week of age and occurs much less frequently when intercat play is apparently peaking around 10–14 weeks of age (19). This is consistent with the suggestion that the expression of behaviours in play may help to refine their later functional expression, at which time their appearance in play may need to decline to avoid ambiguity. For example, Caro (24, 35) in his studies of different forms of play in kittens have suggested that arching and chasing are under the control of the same factors that control later agonistic behaviour and separate to those associated with future predatory behaviour, given the negative relationship in their frequency between 8 and 12 weeks of age.

Cat contact appears to decrease while object contact increases from 7th to 8th week of age (17) supporting the separation of related forms of play. It seems that cats continue to play regularly with conspecifics until about 4 months of their age but their attention is gradually drawn to objects, as their need to obtain food by themselves increases (34). Indeed the presence of prey appears to have an inhibitory effect on intercat play (24), while the provision of meat and object play might reduce predatory behaviour, as indicated by the number of prey carried home (44), further reinforcing the function relationships already described.

This is supported by the observation that some behaviour patterns associated with manipulation with prey such as “paw” and “bite” (24) are similar to those used in manipulation with an object (17). The potential for cats to treat other cats as predatory objects is also supported by the observation that observations of cat approaching, pawing, and biting of another cat show positive correlations with respect to other predatory measures from 8th to 12th week of age alongside holding of another cat (24, 35). Thus predation, predatory play and object play appear to be related and this is consistent with the view of Panksepp (20) that they are all expressions of a SEEKING rather than PLAY system even when used within the context of interaction with another cat. However, striking another cat with a paw and biting may also be seen within the functional context of agonistic behaviour in adult cats (24) which is an expression of RAGE [*sensu* (20)] and so it should not be assumed that any given behaviour is specific to a given motivational or affective system. These observations further support the suggestion that there is no single motivational system controlling play.

Clearly, the dynamics of cat play change over time and intercat play can be used to refine express skills associated with predation and agonistic behaviour. Mutual social play appears to be rarer as the kitten matures and this raises concern over the labelling of adult exchanges as playful on the basis of “common sense.” The older the cats are, the more cautious we need to be about interpreting their social behaviour.

## Metasignals and Intercat Play

Metasignals are used to help clarify how a piece of information (such as a deliberate action) should be understood. Within the context of play, metacommunication concerns the exchange of signals to indicate that what follows is play (45), rather than what should happen within play. Metacommunicative signals from a sender must be unambiguous and reduce distance between interacting animals (46). Potential metasignals indicating a social play context have been studied in dogs (47–49), while there is some debate about their specific meaning and function (40, 50, 51).

It has been suggested that cats, like dogs, can use a play face (mouth slightly open without showing teeth with ears and eyes relaxed or fairly alert) to communicate a distinction between playful and “serious” encounters (10, 11, 16, 34) but this is a somewhat subjective description and to our knowledge this has never been established by scientific observation of domestic cats. The vertical position of a tail during social encounters (tail-up posture) signals affiliative intent of the cat-sender and thus reduces the risk of aggressive behaviour within an intercat interaction (52, 53). It has been suggested that “tail-up” is also used during social and object play (54) together with other tail movements (11) but the significance of tail postures as metasignals during the mutual social play lacks scientific evaluation. Further observational studies should explore this potentially important contribution to intercat communication. Moreover, as lateralisation of the tail might affect willingness to approach in dogs (55), this aspect should be examined in cats as well, as it might further clarify the tail-signalling function in this species.

Although as mentioned above, certain behaviours such as arching and chasing, whose miscommunication could have serious consequences, tend to decline as features of play, they do not disappear and so it is predicted that there should be some metasignal to qualify these actions for the context of mutual social play. Nonetheless, there appears to be a general lack of research on metasignalling in relation to play in cats, despite its potential importance, especially in adult cats (another understudied area of play in cats). As discussed further below, recognising play in adult cats is also an area of practical concern for owners and those seeking to support the welfare of pets.

## Feline Sociality

The study of social play in domestic cats is complicated by the suggestion that they, unlike their ancestral species, are potentially much more social animals, capable of forming social groups (56, 57). The core of the group is typically formed by related individuals (56) but also non-related cats may live amicably when they are familiar with each other for a longer period (58). Cats that belong to the same social group usually express affiliative behaviours such as holding their tails up when approaching each other, rubbing against each other, allogrooming, sleeping in close contact together and it widely believed that such individuals are capable of playing together as well (57, 59).

Suggestions have also been made about the nature of aggressive acts when they form part of intercat play. Such displays should include minimal or no vocalisation such as growling, hissing or screaming, in addition scratching and biting is inhibited (59) and play fighting should include plenty of pauses (60). When rough-and-tumble play gets too rough one cat may terminate it by simply walking away from the interaction (41), however escalation into harmful interaction is a commonly mentioned scenario in the clinical feline behaviour literature (41, 59, 61). However, many of these points remain speculative and untested in the scientific literature, perhaps because of the problem of reliably identifying cat play without creating circular arguments.

## Problems Arising From Research Methodology Aimed at Increasing Our Understanding of Intercat Play

### Observational Studies

The majority of observational research on intercat play has focused on developmental studies in kittens; monitoring spontaneous behaviours of play, from birth until 24th week of age, in the presence of at least one other cat (mother or sibling). This has been conducted in stable (17, 19, 24, 25, 34) or dynamic environmental conditions. These include assessment of the impact of social isolation (62), separation from mother (36), interruption of lactation (37, 63), food rationing (64), and litter size (14) (See Table 2 for an overview of the main findings of these observational studies). Time of day designated for observation, duration of observation and sampling techniques differed among studies. In nearly all studies [the single exception being (34)], laboratory cats were observed and so how this relates to what emerges in the more complex home environment of most cats is questionable. The studies show what can affect play behaviour not what necessarily does in the typical world setting.

**Table 2:** Overview of main findings from observational studies of intercat play.

**Reference**	**Context in which intercat play has been studied**	**Main findings related to intercat play**
West (34)	Development of intercat play	Eight behaviours of intercat play and their sequences were identified. Intercat play was most frequent in period from 4 weeks to 4 months of cat's age.
Barrett and Bateson (17)	Development of play	Object contact, Wrestle, and Stalk increased and Cat contact and Arch decreased from 4–7 week period to 8–12 week period, suggesting existence of few controlling systems of play behaviour category.
Caro (35)	Relationship between kitten behaviour and adult predation	Approaching, pawing, holding, and biting were positively correlated with adult predatory behaviour and attention to prey, while rearing, arching, and chasing showed negative correlation in this relationship.
Guyot et al. (62)	Effects of social isolation on behaviour of young cats	Kittens deprived of littermates since birth or 2 days of age were less successful in maintaining non-hurtful character of intercat play when tested socially from 8th week to 20th week of age.
Bateson et al. (63)	Effects of lactation interruption (in 5th week after birth) on play in kittens	Interrupted lactation in 6th week after the birth, and thus earlier weaning, resulted in higher frequency of object play but have not influenced intercat play.
Bateson and Young (36)	Effect of separation from mother on development of play in cats	Separation from mother in 5th week after birth resulted in higher frequency of intercat play in period from 5th to 7th week of age of kittens.
Caro (24)	Relationship between intercat play and development of predatory behaviour	All measures of intercat play, with exception of arching, increased in frequency from 5th to 8th week. In the period from 8th to 12th week approaching, pawing and biting were more closely associated with predatory behaviour and rearing arching and chasing became less associated with predation.
Caro (25)	Influence of sex on termination of intercat play	Males from all-male groups played together more than females from all-male groups in period from 12 to 16 weeks of age, while frequencies of females' play behaviours declined with decreasing number of males in group.
Martin and Bateson (37)	Effects of lactation interruption (in 4th week after birth) on play in kittens	Early weaned kittens showed higher frequencies of intercat play than kittens from control group.
Mendoza and Ramirez (19)	Relationship between play and cohesion and aggression in cats	Occurrence of intercat play peaked between 9th and 14th week, period during which cohesion behaviours (approach, physical contact, interindividual closeness, nose-nose contact) were observed.
Mendl (14)	Effects of litter-size variation on development of play in cats	Single kittens experienced less intercat play than kittens with siblings but directed play behaviour more on their mothers, which did not always reciprocate this activity.
Bateson et al. (64)	Effect of lactating mother's food rationing on play in kittens	Frequency of intercat play did not differ between kittens from rationed families and those from *ad libitum* families.

Another group of observational studies concern simple descriptions of intercat play in adult cats (65–67), which often lack useful controls and may define social play very loosely. This can lead to confusion about the meaningful characteristics of social play. For example, one study, supposedly on social play, focused only on play between a cat and human in domestic settings and did not consider who initiated the action and how this might affect the behaviours observed (21). Another considered two cats playing with the same toy simultaneously as a form of social play (23). With poor definition of “social play,” the assumption that it is an indicator of good welfare may be challenged and the validity of the conclusions drawn, especially the absence of an effect of an intervention, may be questionable.

### Questionnaire Based Studies

To our knowledge, somewhat surprisingly, the structure of intercat play as a specific entity has not been studied using questionnaire based studies. However, “Playfulness” in the form of a single item on play with other household cat(s) together with 13 other items on playful behaviour related to object and self play, does form part of the Feline Behavioural Assessment and Research Questionnaire (Fe-BARQ) (68, 69). This appears to be the only validated questionnaire of relevance developed to date. Fe-BARQ consists of 101 items relating to the behaviour of cats which group into 23 factors; each item is scored using a 5-point Likert scale referring to the frequency of behavioural item (0 = never to 4 = always). In relation to intercat play, Fe-BARQ combines this item with other play contexts (e.g., play with object or people) into a common “Playfulness factor,” because the former is more closely related to the latter than any other aspect of behaviour assessed. This does not mean that it shares a common mechanistic basis and the concept of “playfulness” as a common factor may be misleading, as demonstrated by some of the specific relationships identified in section on the structure of intercat play, above. Mindful of this limitation, Fe-BARQ has been used in a recent study (70) to evaluate the relationship between aggression toward other cats and playfulness with objects or people and the item relating to “social play” (cat plays with other household cat/s). This found a negative relationship between “social play” and intraspecific aggressivity and a positive relationship with other forms of playfulness, but neither relationship was strong. This supports our suggestion that there is a fair degree of independence between social play and these other factors.

## Concluding Comments

Burghardt's five criteria for recognising play are difficult to apply scientifically when two cats are playing together, and there is a danger that circular reasoning is applied when analysing play behaviour in cats. Accordingly, the descriptive value of observational field and contextual data needs to be clearly separated from the functional inferences which it may be used to support. The latter are hypotheses about the likely emotional-motivational state of the two interacting cats, and should be considered tentative until we can apply more definitive tests. Appealing to common sense is inadequate. In order to make progress, it is important that a standard terminology is adopted and the distinction between the observed and the inferred is clearly acknowledged and articulated. A functional classification is important from a clinical behavioural context, where the humane management of the behaviour, and thus hypothesised internal state, is important. Indeed, it might be that through careful analysis of intervention programmes and the gathering of detailed ethological data, in line with the recommendations above, that we can test our hypotheses and advance our understanding of whether “these two cats are playing” in a scientifically more rigorous way.

We propose here adoption of a standard terminology and functional affective classification to play between cats considering emotion and motivation. Thus, a cat may be playing by itself, or be with another and perceiving it as an object (including prey), in which case the activity relates to the desire to learn about an individual's own capacity in relation to the physical environment; this should not be considered from a functional perspective to be social play, even if another cat is involved (i.e., it is a form of intercat play). At other times interaction with another cat may facilitate learning about the individual's capacity in relation to the social environment (including both social skills and social role), and in these circumstances we would argue that the interaction is from a functional perspective social play, which may or may not be mutual. Further it needs to be recognised that bouts of intercat interaction can start as mutual social play but can turn into intercat play, when reciprocity is lost or the interaction becomes truly agonistic. Such alternation between emotional-motivational states is not uncommon in cats and adds a layer of complexity not evident in some other species, such as the dog.

## Author Contributions

NGK and DM conceptualised and designed the manuscript. NGK reviewed the literature. All authors contributed to the article and approved the submitted version.

## Acknowledgements

We are particularly grateful to Mr. Vincent Kmec for his wonderful illustrations, which are presented in the figures of this article, and thus for his precious help in the visualisation of different intercat play behaviours.

## Conflict of Interest

The authors declare that the research was conducted in the absence of any commercial or financial relationships that could be construed as a potential conflict of interest.

## Publisher's Note

All claims expressed in this article are solely those of the authors and do not necessarily represent those of their affiliated organizations, or those of the publisher, the editors and the reviewers. Any product that may be evaluated in this article, or claim that may be made by its manufacturer, is not guaranteed or endorsed by the publisher.
